# The Ethanolic Extract of *Caesalpinia sappan* Heartwood Inhibits Cerebral Ischemia/Reperfusion Injury in a Rat Model Through a Multi-Targeted Pharmacological Mechanism

**DOI:** 10.3389/fphar.2019.00029

**Published:** 2019-02-05

**Authors:** Yan-Jun Wan, Li Xu, Wen-Ting Song, Yu-Qi Liu, Li-Chao Wang, Ming-Bo Zhao, Yong Jiang, Lian-Ying Liu, Ke-Wu Zeng, Peng-Fei Tu

**Affiliations:** ^1^State Key Laboratory of Natural and Biomimetic Drugs, School of Pharmaceutical Sciences, Peking University, Beijing, China; ^2^Institute of Basic Medical Sciences, Xiyuan Hospital, China Academy of Chinese Medical Sciences, Beijing, China; ^3^College of Materials Science and Engineering, Beijing University of Chemical Technology, Beijing, China

**Keywords:** *Caesalpinia sappan* L., middle cerebral artery occlusion, oxygen-glucose deprivation/reperfusion, neuroprotection, multi-target mechanism

## Abstract

**Background:** *Caesalpinia sappan* L. (*C. sappan*) is a traditional Chinese medicinal plant. The dried heartwood of *C. sappan* (also known as *Sappan* wood) has been widely used for the folkloric medical treatment of ischemic cerebral stroke in China. However, the detailed underlying pharmacological mechanism still remains largely unexplored.

**Methods:** In this study, a middle cerebral artery occlusion (MCAO) rat model was employed to elucidate the mechanism of the anti-cerebral ischemic effects of *C. sappan* ethanolic extract (CEE). Moreover, systemic multi-target identification coupled with gene ontology biological process (GO BP) and reactome pathway analysis was used to investigate the potential neuroprotective mechanism. Furthermore, the presumed mechanism was confirmed through biological analysis by determining the effects of CEE on the identified signaling pathways in PC12 cells model-induced by oxygen-glucose deprivation/reperfusion (OGD/R).

**Results:** Our study demonstrates that CEE (both through *in vivo* administration at a dosage of 300 mg/kg and through *in vitro* incubation at a dosage of 2.4 μg/mL) is a neuroprotective agent that can effectively inhibit neuronal damage, promote synaptic generation, and suppress the activation of neutrophils, microglia, and astrocytes. Moreover, the neuroprotective mechanism of CEE is mediated via regulating 150 potential target proteins, which are associated with 6 biological processes and 10 pathways, including JAK-STAT, HSP90 and DNA damage/telomere stress.

**Conclusion:** CEE can exert neuroprotective effect through multi-target pharmacological mechanisms to prevent ischemia/reperfusion-induced cerebral injury.

## Introduction

Ischemic cerebral stroke, also known as cerebral infarction, makes up approximately 80% of all strokes with a high risk of mortality (Dabrowska-Bender et al., 2017; Wang W. et al., 2017). The mechanisms that are associated with ischemic cerebral stroke are complex and mainly include neuronal excitotoxicity, disequilibrium of energy metabolism, oxidative stress, inflammation and cell apoptosis (Sims and Muyderman, 2010). These pathophysiologic processes overlap and intercommunicate to form a vicious cycle, which results in irreversible brain damage and persistent neurologic dysfunction. Two basic approaches, namely focal cerebral blood flow recovery and direct neuroprotection, have emerged as the treatment of ischemic stroke. However, to date, no other novel strategies have proven to be efficacious and safe in clinical trials (Brouns and De Deyn, 2009).

Over years of research, the multi-target approach for ischemic stroke therapy has drawn the close attention of many investigators. TCM, which is recognized as a key source for the discovery of drugs with complex chemical compositions, is characterized by multiple targets. *Caesalpinia sappan* L. is a traditional medicinal plant with dried heartwood that is used for the promotion of blood circulation and removal of blood stasis, according to Chinese Pharmacopoeia (National Pharmacopoeia Committee, 2015). In particular, among the local populations in China, *C. sappan* has been extensively used in the treatment of ischemic stroke. Additionally, previous pharmacological studies have also shown that some of the chemical constituents of *C. sappan* produce neuroprotective effects through anti-neuronal apoptotic (Zeng et al., 2015a), anti-inflammatory (Jia et al., 2016) and anti-thrombotic (Islam et al., 2016) activities. However, these previous investigations did not attempt to identify the direct molecular targets which would systematically uncover the detailed pharmacological mechanisms of *C. sappan*-mediated cerebral protection.

In the present study, we attempted to identify the direct target proteins of *C. sappan* ethanolic extract (CEE) with CEE beads from the SH-SY5Y cell lysate, followed by LC-MS/MS analysis. Moreover, the potential neuroprotective mechanism was investigated using bioinformatics analysis, including GO BP and reactome pathway enrichment analysis, as well as biochemical verification. Collectively, this study revealed the underlying multi-target pharmacological mechanism that gives rise to the neuroprotective action of CEE.

## Materials and Methods

### Preparation of the Ethanolic Extract of the Heartwood of *C. sappan* L. (CEE)

The heartwood of *C. sappan* L. was purchased from Anguo Medicinal Materials Market (Hebei Province, China). Botanical identification was performed by Prof. Peng-Fei Tu from the School of Pharmaceutical Sciences, Peking University. The plant material was dried and powdered. The coarsely powdered sample was extracted by refluxing with 70% ethanol (1:8, w: v) for 1 h. This extraction process was repeated three times and the obtained extract was filtered. The filtrate was dried under reduced pressure, finally yielding a dark reddish-brown powder.

### Phytochemical Analysis

The phytochemical constituents of CEE were analyzed using an Agilent 1600 series HPLC (Agilent Technologies, CA, United States), and compared to the reference compounds brazilin and protosappanin B (Chengdu Desite, Sichuan, China). The analytical column that was used was an Agilent Zorbax SB-Aq C_18_ column (4.6 mm inner diameter × 250 mm length, 5 μm particle size), which was maintained at 25°C. The mobile phase consisted of water (A) and acetonitrile (B). The following gradient elution program was used: 0–30 min, 5–10% B; 30–45 min, 10–16% B; 45-60 min, 16–18%; 60–90 min 18–23%; 90–105 min, 23–30%; 105–120 min, 30–40%. The analysis was performed at a flow rate of 0.8 mL/min with the UV detection wavelength at 285 nm (Chu et al., 2013). CEE and the reference compounds (brazilin and protosappanin B) were dissolved in acetonitrile. After filtration through a 0.25-μm membrane, a 10-μL aliquot was injected into the HPLC for analysis. The identities of the resulting main chromatographic peaks were confirmed by comparing the retention times and UV spectra with those of the reference standards.

### Experimental Animals

Sprague-Dawley (SD) rats (6–8 weeks old, weighing 230–250 g) were obtained from the Department of Laboratory Animal Science (Peking University Health Science Center) and were housed under a 12 h/12 h light/dark cycle at 25 ± 2°C. Animal care and experimental protocols were performed based on ‘Detailed Rules and Regulations for Administration and Implementation of Biomedical Animal Experiments’ (No. 1998-55, Ministry of Public Health, China). These protocols were approved by the Institutional Committee on Animal Care and Use, Peking University.

### Establishment of Rat Middle Cerebral Artery Occlusion (MCAO) Model

The MCAO model was established in accordance with the Longa line embolism method, with minor modifications (Longa et al., 1989). Following intraperitoneal anesthesia with 2% pentobarbital sodium (30 mg/kg), a cervical median incision was made in each of the rats. The right vessels, including the CCA, ECA, ICA, and PPA, were then isolated. Subsequently, the ICA was occluded by 24/34, 26/36 or 26/38 model filament (0.24–0.26 mm diameter, 0.34–0.38 mm length) coated with poly-L-lysine. The appropriate filament was selected, based on the body weight of the rat, and inserted into the ICA until the blood supply of the middle cerebral artery was blocked. After occlusion for 1.5 h, the filament was carefully withdrawn to induce reperfusion. Then the cervical incision was sutured. During the surgery, the rectal temperature was maintained at 37 ± 0.5°C. The rats in the sham group were treated similarly as the MCAO rats, though the filament was not inserted.

### Drug Administration

The rats were randomly divided into six groups (*n* = 15 per group), including (i) the sham group, (ii) the model group, (iii) group treated with 30 mg/kg CEE, (iv) group treated with 100 mg/kg CEE, (v) group treated with 300 mg/kg CEE, and (vi) group treated with 20 mg/kg GND (as a positive control) (Xu and Lee, 2004; Shen et al., 2007; Tang et al., 2017). After MCAO model established, the indicated drugs were administered immediately. Because of intragastric administration often induces gastric regurgitation and choking to death under anesthesia. So the rats in drug treatment groups were administered intraduodenally with the indicated agents dissolved in normal saline. The sham and model groups received equal volumes of saline. The drugs were administered only once. Rats died were excluded from the study.

### Evaluation of Neurological Deficits

The rats were tested for neurological deficits 24 h after MCAO (*n* = 15 per group), based on the Zea-Longa scoring system (Longa et al., 1989) as follows: score 0, no apparent neurological deficits; score 1, unable to extend the affected forelimb; score 2, walking in circles; score 3, tumbling to the side while walking because of hemiplegia; and score 4, unconscious and unable to walk.

### Measurement of Infarct Volumes

Six rats per group were anesthetized with pentobarbital and sacrificed after neurological assessment. The brains were removed and cut into five sections approximately 2 mm thick. The slices were placed in a petri dish containing 0.05% TTC at 37°C for 30 min, followed by immersion in 10% formaldehyde. The slices were photographed using a CCD camera connected to a computer, and quantified using the image analyzing software (Image J 1.46r). The infarct area of each slice was calculated as follows: infarct area = measured infarct area × (1- [ipsilateral hemisphere area-contralateral hemisphere area]/contralateral hemisphere area).

### Transmission Electron Microscope (TEM) Analysis

Six rats in each group were anesthetized with pentobarbital and perfused with 0.9% sodium chloride followed by 2% phosphate buffered glutaraldehyde and 4% phosphate buffered paraformaldehyde. Three cubes of the representative brain tissues approximately 1–2 mm on lesion hemisphere, were prepared. Each tissue sample was fixed in 1% osmium tetroxide, dehydrated in graded acetone, and embedded in Epon 812. Semithin (0.5 μm) sections were cut from tissue blocks and stained with 0.5% toluidine blue. The sections were then stained with 0.25% lead citrate and 5% uranyl acetate in 50% methanol, and observed with a JEOL 100CX electron microscope (Hitachi HT7700-SS; Hitachi, Tokyo, Japan).

### Brain Slice Preparation

Three rats in each group were anesthetized with pentobarbital and perfused with 0.9% sodium chloride followed by 4% phosphate buffered paraformaldehyde. The brains were removed and fixed in 4% phosphate buffered paraformaldehyde, dehydrated in a series of ethanol, and embedded in paraffin. Coronal sections of 5 μm thickness were cut for assay.

### Histological Assessment

The slices were deparaffinized and rehydrated, stained with 0.1% cresyl violet-luxol (Nissl staining), hematoxylin-eosin (HE staining) or xylidine-ponceau (Masson staining). Sections were observed under the bright-field mode with a microscope (IX73, Olympus, Japan).

### TdT-Mediated dUTP-Biotin Nick-End Labeling (TUNEL) Assay

The TUNEL staining was performed according to the manufacturer’s instructions (Roche Molecular Biochemicals, Inc., Mannheim, Germany). Briefly, the slices were deparaffinized and rehydrated, treated with proteinase K (20 μg/mL) for 15 min and with 3% hydrogen peroxide for 30 min. Following this, the cortices of lesion hemisphere sections were incubated with TdT at 37°C for 1 h and photographed under fluorescence mode with a microscope (IX73, Olympus, Japan).

### Immunohistochemical (IHC) Assay

For IHC assay, the slices were deparaffinized and rehydrated, submerged into citrate buffer for 20 min, treated with 3% hydrogen peroxide for 30 min and incubated with 5% normal goat serum for 60 min. Then, the sections were incubated with anti-CD11b/c, anti-Iba-1, anti-GFAP, or anti-MAP-2 antibody (1:100) overnight at 4°C. After washed with PBST (PBS+1% tween), the sections were incubated with peroxidase-conjugated antibody (1:1000) at 37°C for 30 min. The immunoreactivity was visualized by incubating sections with DAB for 2 min, and performing counterstaining with hematoxylin. Finally, the cortices of lesion hemisphere were photographed under the bright-field mode with a microscope (IX73, Olympus, Japan).

### Cell Culture

Human bone marrow neuroblastoma (SH-SY5Y) cells and rat adrenal pheochromocytoma (PC12) cells were obtained from Peking Union Medical College Cell Bank (Beijing, China). These cells were cultured in 4.5 g/mL glucose in DMEM medium supplemented with 10% fetal bovine serum, 100 μg/mL penicillin, and 100 μg/mL streptomycin, and were maintained in a humidified incubator with 95% air and 5% CO_2_ at 37°C.

### Identification of Direct Target Proteins of CEE

Based on a previously reported method (Wan et al., 2017; Zeng et al., 2017), we successfully prepared CEE beads (see Extended Experimental Procedure and Supplementary Figures S2–S4). The CEE beads were incubated with SH-SY5Y cell lysate proteins (0.8 mg) at 4°C for 12 h to capture all of the target proteins. In the meantime, the control beads were incubated with lysate as negative control to avoid false positive results. The bead-captured proteins were then separated using 10% SDS-PAGE and visualized by silver staining. The corresponding proteins were prepared by in-gel digestion and identified by LC-MS/MS (Shevchenko et al., 2006). The valuable simple peptide sequences detected by nano-HPLC-tandem LTQ Velos pro mass spectrometer (Orbitrap Velos Pro; Thermo Fisher Scientific, Waltham, MA, United States) were retrieved by UniProt, and a total of 150 potential binding target proteins of CEE were identified (Supplementary Table S1). Four target proteins were selected for verification by western blot assay using specific antibodies.

### Gene Ontology Biological Process (GO BP) and Reactome Pathway Enrichment Analysis

All of the potential target proteins were analyzed through pharmacological mechanism exploration by Cytoscape 3.4.0 (Cytoscape Consortium, United States) software. The analysis was based on the *Homo sapiens* gene annotation databases, with more than two genes in each term and a *P*-value < 0.05.

### Cell Viability Assessment

Cell viability was assessed by the MTT assay. CEE power was dissolved in water and was adjusted to final concentrations ranging from 0.3 to 19.2 μg/mL by diluting with the growth medium. PC12 cells were seeded at the initial density of 0.5 × 10^4^ cells/well in 96 well plates for 24 h and exposed to indicated concentrations of CEE for 24 h. MTT stock solution in full culture medium was added to each well to attain a final concentration of 0.5 mg/mL. Plates were incubated for 4 h at 37°C under normoxic conditions. The medium with MTT was then removed and 0.1 mL of DMSO was added to each well and incubated for 10 min to dissolve the formazan crystals. The absorbance was read at 570 nm using a microplate reader.

### Oxygen-Glucose Deprivation/Reperfusion (OGD/R) Model

PC12 cells were washed with PBS twice and cultured in Earle’s balanced salt solution (116 mM NaCl, 5.4 mM KCl, 0.8 mM MgSO_4_, 1 mM NaH_2_PO_4_, and 0.9 mM CaCl_2_) in an oxygen-free incubator (95% N_2_ and 5% CO_2_) for 4 h. Following this OGD incubation, the cells were returned to full culture medium under normoxic conditions for 2 and 24 h, respectively. Control cells were incubated in a regular cell culture incubator under normoxic conditions for the same duration.

### Hoechst 33258 Staining and AO/EB Staining

PC12 cells were seeded at the initial density of 5 × 10^4^ cells/well in 24 well plates and subjected to OGD. For Hoechst 33258 staining, the cells were fixed with 4% paraformaldehyde for 15 min and washed twice with PBS, followed by staining with Hoechst 33258 working solution (1 μg/mL) for 30 min at room temperature in the dark. These cells were observed with a fluorescence microscope (IX73, Olympus, Japan) at the excitation wavelength of 350 nm and the emission wavelength of 461 nm. For AO/EB staining, no fixation was performed, the cells were washed twice with PBS and treated with the AO/EB mixture (2 μg/mL) for 2 min at room temperature in the dark. These cells were visualized using a fluorescence microscope (IX73, Olympus, Japan) at the excitation wavelength of 490 nm and the emission wavelength of 530 nm for AO staining, and at the excitation wavelength of 520 nm and the emission wavelength of 590 nm for EB staining.

### Western Blotting

PC12 cells were cultured in 6 well plates with 20 × 10^4^ cells/well for western blotting examination. Immediately following OGD/R, the cells were rinsed with ice-cold PBS and collected. The total proteins were extracted using RIPA buffer (50 mM Tris, pH 7.4, 150 mM NaCl, 1% NP-40, 0.5% sodium deoxycholate, 0.1% SDS, 1 mM EDTA) with cocktail protein inhibitors and centrifuged at 12,000 *g* for 15 min at 4°C. Equal amounts of protein samples (50 μg) were loaded in each lane and proteins were resolved by 8–15% SDS/PAGE. The proteins were then electro-transferred onto a PVDF membrane using a wet transfer system. The membranes were blocked in 5% no-fat milk for 1 h at room temperature and then incubated with primary antibodies (1:1000, Cell Signaling Technology) overnight at 4°C. Following this, the membrane was incubated with anti-rabbit secondary antibody conjugated to horseradish peroxidase (1:2000, Cell Signaling Technology) for 2 h. The membranes were washed and visualized by an enhanced chemiluminescent substrate and scanned with the Kodak Digital Imaging System (5200 Multi, Tanon, China).

### Statistical Analysis

The data were analyzed using SPSS 20.0 software (SPSS, Chicago, IL, United States) and expressed as mean ± SD. Statistical comparisons between different treatments were made using Shapiro–Wilk normality for normal distribution and tested for homogeneity. One-way analysis of variance with LSD *post hoc* test was performed if there was homogeneity. A non-parametric test with Bonferroni’s *post hoc* test was performed if there was no homogeneity. Statistical significance was set at *P* < 0.05.

## Results

### CEE Reversed MCAO-Induced Cerebral Injury in Rats

**FIGURE 1:**
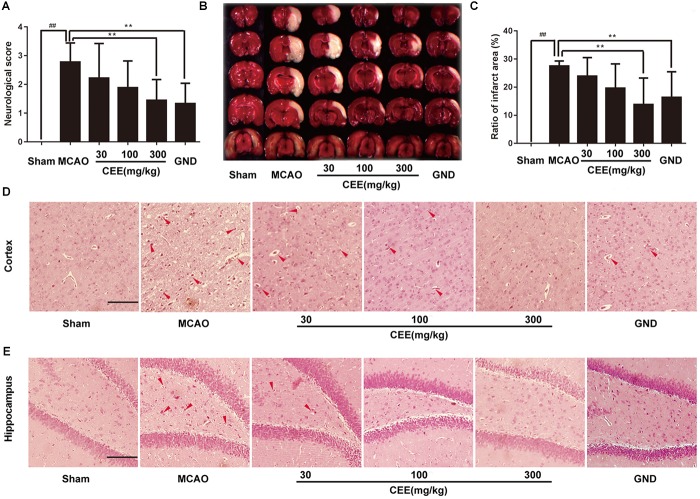
The neuroprotective effect of *Caesalpinia sappan* ethanolic extract (CEE) in the rat MCAO model. **(A)** The neurological deficit score was evaluated after 1.5 h ischemia and 24 h reperfusion (*n* = 15). **(B)** The cerebral infarction size as determined through TTC staining (*n* = 6). **(C)** The percentage of ischemic lesion area, calculated using the ratio of the infarction area to the whole slice area (*n* = 6). **(D)** Histological changes in the cerebral cortex region as detected by HE staining assay (Scale bar: 50 μm). **(E)** Histological changes of cerebral hippocampal region as detected through HE staining assay (scale bar: 50 μm). Three rats in each group and three slices for each rat were examined for HE staining assay. Data are expressed as the mean ± *SD*. ^##^*P* < 0.01 vs. sham group, ^∗∗^*P* < 0.01, ^∗^*P* < 0.05 vs. MCAO group.

Through HPLC analysis, a total of 10 ingredients were detected in CEE. Brazilin and protosappanin B were two of the major chemical ingredients in CEE. The HPLC characteristic spectra of CEE and the standards (i.e., brazilin and protosappanin B) are shown in Supplementary Figure S1. To determine the CEE-mediated neuroprotective effect against ischemia/reperfusion-induced cerebral injury, we investigated the neurological deficits discernible in the rats. Compared with the sham group, MCAO rats exhibited significant neurological deficits, which were effectively reversed by CEE treatment (Figure 1A). The area of cerebral infarction was then evaluated. As shown in Figure 1B,C, no infarction was observed in the sham group; however, 24.94–29.98% ipsilateral cerebral infarction was found in the MCAO group. The area of cerebral infarction was significantly lower in the CEE treated animals than in the MCAO animals, the reduction in the area of cerebral infarction occurred in a dose-dependent manner. To further investigate the effect of CEE on the morphological features of neurons, we performed HE staining analysis. The results showed that MCAO induced obvious neuronal damage, including abnormal nuclei and neuronal shrinkage in the cerebral cortex and cerebral hippocampal regions. After CEE treatment, the number of shrunken cells was significantly lower in the cerebral cortex (Figure 1D) and cerebral hippocampal (Figure 1E) regions than in the MCAO group. We found that GND treatment as a positive control, showed similar neuroprotection against the MCAO insult. Taken together, our data show that CEE effectively reduced acute cerebral ischemia/reperfusion-induced injury *in vivo*.

### CEE Inhibited Neuronal Apoptosis in MCAO Rats

**FIGURE 2:**
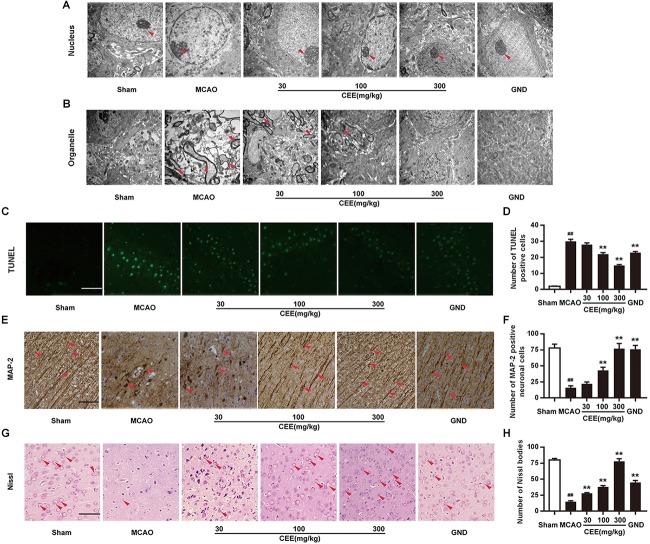
*Caesalpinia sappan* ethanolic extract inhibits neuronal apoptosis in MCAO rats. **(A)** Ultrastructural changes in the neuronal cell nuclei in brains as detected by TEM (scale bar: 3 μm). **(B)** Ultrastructural changes of the neuronal cell organelles, including the endoplasmic reticulum and mitochondria, in brains as detected by TEM (scale bar: 3 μm). **(C)** Neuronal apoptosis as detected by TUNEL staining assay (scale bar: 50 μm). **(D)** Quantitative assessment of TUNEL positive cells in rat brain cortex penumbra per field. **(E)** MAP-2 expression as detected by IHC assay using a specific antibody for MAP-2 (scale bar: 50 μm). **(F)** Quantitative assessment of MAP-2 positive neuronal cells in rat brain cortex penumbra per field. **(G)** Morphologic changes of neuronal synapses as detected by Nissl staining assay (scale bar: 50 μm). **(H)** Quantitative assessment of Nissl bodies in rat brain cortex penumbra per field. Six rats in each group and three slices for each rat were examined for TEM analysis. Three rats in each group and three slices for each rat were examined for TUNEL, Nissl staining and IHC assay. Data are expressed as mean ± *SD*. ^##^*P* < 0.01 vs. sham group, ^∗∗^*P* < 0.01, ^∗^*P* < 0.05 vs. MCAO group.

First, TEM analysis was employed to explore the effect of CEE on ultrastructural cerebral changes. In the sham group, the nuclear membranes possessed integrity with normal subcellular structures, and the mitochondria were present in long and tubular networks with obvious ridges. In the MCAO group, the neurons showed evident edema and abnormal chromatin distribution. The mitochondria were no longer intact and the cristae were not visible. However, after CEE treatment, the shape of the neuronal nuclei was markedly recovered (Figure 2A), and the appearance of the mitochondrial was also significantly improved (Figure 2B). Additionally, cell apoptosis was determined by TUNEL staining. Obvious TUNEL-positive cells were observed in the MCAO group. However, in the CEE treated group the number of TUNEL-positive cells was significantly lower than in the MCAO group (Figure 2C,D). These results illustrate that cell apoptosis in MCAO-induced cerebral injury in rats was significantly suppressed by CEE treatment.

Microtubule-associated protein-2 (MAP-2) is mainly present in mature neurons and is selectively concentrated at neuronal synapses. In this study, MAP-2 immunoreactivity was also applied to delineate the status of the synaptic generation. In the sham group, MAP-2-positive subcellular structures were extensively observed. However, the number of MAP-2-positive subcellular structures was significantly lower in the MCAO group than in the control group. This change was markedly reduced in the CEE treated group (Figure 2E,F). Furthermore, Nissl bodies are crucial sites for protein synthesis in neurons. Here, the Nissl staining assay clearly showed that Nissl bodies were missing in the MCAO group. This phenomenon was effectively rescued by CEE treatment, suggesting that neurons are protected by the action of CEE in the MCAO model Figure 2G,H).

### CEE Inhibited Neutrophil and Microglial Activation in MCAO Rats

**FIGURE 3:**
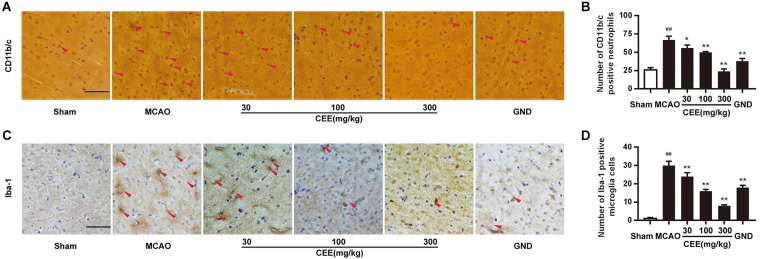
*Caesalpinia sappan* ethanolic extract inhibits neutrophil and microglial activation in MCAO rats. **(A)** CD11b/c expression in brains as detected by immunohistochemical staining assay using a specific antibody for CD11b/c (scale bar: 50 μm). **(B)** Quantitative assessment of CD11b/c positive neutrophils in rat brain cortex penumbra per field. **(C)** Iba-1 expression in brains as detected by immunohistochemical staining assay using a specific antibody for Iba-1 (scale bar: 50 μm). **(D)** Quantitative assessment of Iba-1 positive microglial cells in rat brain cortex penumbra per field. Three rats in each group and three slices for each rat were examined for IHC assay. Data are expressed as mean ±*SD*. ^##^*P* < 0.01 vs. sham group, ^∗∗^*P* < 0.01, ^∗^*P* < 0.05 vs. MCAO group.

CD11b/c is an indicator of active neutrophils. Therefore, we investigated the activation of neutrophils by detecting the expression of CD11b/c in rat brains with the IHC assay. As shown in Figure 3A,B, the expression of CD11b/c-positive neutrophils was widespread in the cortex of the MCAO rats. The number of CD11b/c-positive cells was significantly lower in the CEE treated group than in the MCAO group. The Iba-1 protein is a widely recognized marker of microglia. Thus, Iba-1 immunoreactivity was used to demonstrate microglial activation. In the sham group, the microglia were thin and presented with ramified branches. However, in the MCAO group, the microglia were round and had a reduced number of branches. The changes were significantly inhibited by CEE treatment (Figure 3C,D).

### CEE Suppressed Astroglial Activation and Collagen Deposition in MCAO Rats

**FIGURE 4:**
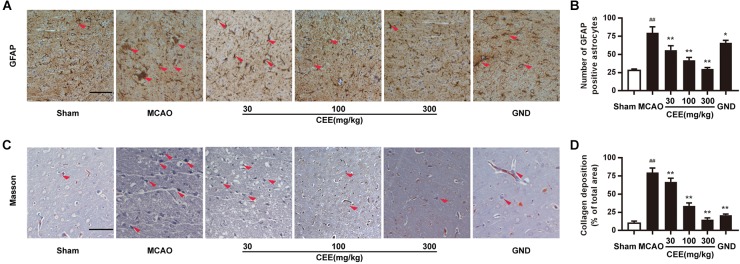
*Caesalpinia sappan* ethanolic extract suppresses astroglial activation and collagen deposition in MCAO rats. **(A)** GFAP expression in brains as detected by immunohistochemical staining assay using a specific antibody for GFAP (scale bar: 50 μm). **(B)** Quantitative assessment of GFAP positive astrocytes in rat brain cortex penumbra per field. **(C)** Collagen deposition as determined through Masson staining assay (scale bar: 50 μm). **(D)** Quantitative assessment of collagen deposition area in rat brain cortex penumbra per field. Three rats in each group and three slices for each rat were examined for Masson staining and IHC assay. Data are expressed as mean ±*SD*. ^##^*P* < 0.01 vs. sham group, ^∗∗^*P* < 0.01, ^∗^*P* < 0.05 vs. MCAO group.

Astroglial activity is mainly characterized by the expression of GFAP. Therefore, GFAP immunoreactivity was employed to demonstrate astroglial activation. We observed that the number of GFAP-positive astrocytes was dramatically increased in the MCAO group (Figure 4A,B). However, CEE treatment markedly down-regulated the number of GFAP-positive astrocytes, suggesting that CEE could effectively suppress the pathological astroglial proliferation under ischemic conditions. Masson staining is usually applied to investigate collagen deposition. In this study, we found obvious collagen deposition in the brains of the MCAO group. However, collagen deposition was significantly attenuated by CEE treatment (Figure 4C,D).

### Direct Target Proteins Identification and Neuroprotective Pathway-Gene Network Analysis of CEE

**FIGURE 5:**
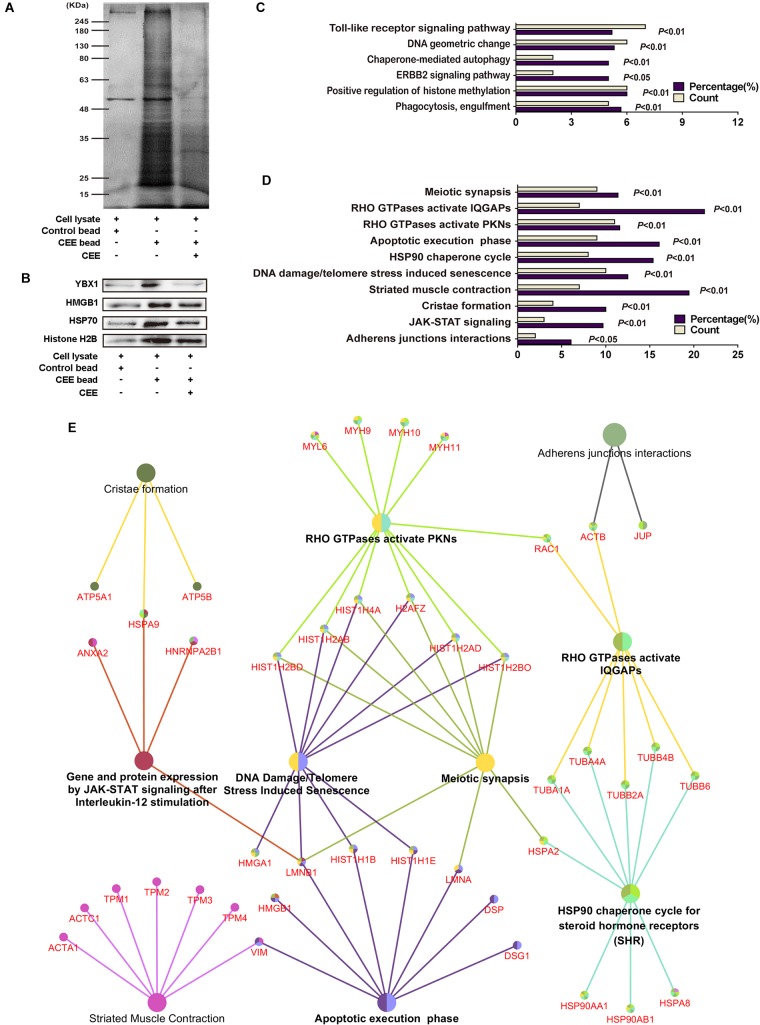
Direct target protein identification and neuroprotective pathway-gene network analysis of CEE. **(A)** The potential target proteins were captured by the CEE-coated beads, separated by 10% SDS-PAGE, and then visualized by silver staining. **(B)** The expression of YBX1, HMGB1, HSP70, and Histone H2B were detected by western blotting. **(C)** 150 potential target proteins were identified by LC-MS/MS and classified into 6 GO terms in biological processes. **(D)** 150 potential target proteins were classified into 10 terms in reactome pathways. **(E)** The pathway-gene network comprised of 150 potential target proteins. The analysis was based on the *Homo sapiens* gene annotation databases, more than two genes in each term and *P*-value < 0.05.

To explore the potential target proteins of the neuroprotective activity of CEE, we attempted to capture direct target proteins using the technology of small-molecular affinity chromatography. As shown in Figure 5A, we discovered 150 potential target proteins for CEE chemical composition-binding. The four selected target proteins that were confirmed by western blot assay were consistent with the data (Figure 5B). The 150 potential target proteins were classified into 6 GO terms in biological processes (*P* < 0.05) and 10 terms in reactome pathways (*P* < 0.05; Figure 5C,D). Particularly, we found that there were six crucial signaling pathways which were heavily involved in the CEE-mediated pathway-gene network, including JAK-STAT, DNA damage/telomere stress, HSP90, apoptotic execution phase, meiotic synapsis and RHO GTPase (Figure 5E).

### CEE Protected OGD/R-Induced PC12 Cell Injury *in vitro*

**FIGURE 6:**
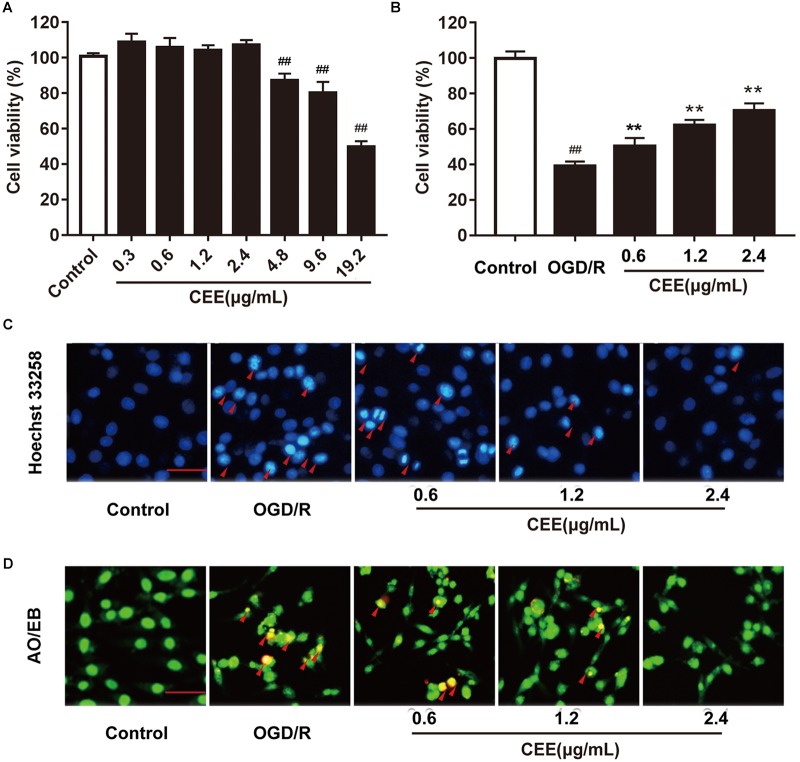
*Caesalpinia sappan* ethanolic extract protects OGD/R-induced PC12 cell injury *in vitro*. **(A)** The effect of CEE on PC12 cell viability. **(B)** CEE increases PC12 cell viability following OGD/R-induced injury. **(C)** The anti-apoptosis effect of CEE on OGD/R-induced PC12 cells as evaluated through a Hoechst 33258 staining assay (scale bar: 20 μm, *n* = 3). **(D)** AO/EB staining assay for the anti-apoptotic effect of CEE on OGD/R-induced PC12 cells (scale bar: 20 μm, *n* = 3). Data are expressed as mean ±*SD*, *n* = 6. ^##^*P* < 0.01, ^#^*P* < 0.05 vs. control group, ^∗∗^*P* < 0.01, ^∗^*P* < 0.05 vs. OGD/R group.

The MTT assay showed that CEE did not influence cell viability at concentrations lower than 4.8 μg/mL (Figure 6A). Therefore, we investigated the effect of CEE on PC12 cells at a concentration range of 0.6–2.4 μg/mL. As shown in Figure 6B, OGD/R induced an obvious decrease in cell viability, which was significantly reversed by CEE treatment in a concentration-dependent manner (Figure 6B). Moreover, in order to explore the effect of CEE on OGD/R-induced cell apoptosis, we also performed Hoechst 33258 and AO/EB staining assays. The results suggest that OGD/R induced a marked increase in the number of apoptotic PC12 cells, which was effectively reversed by CEE treatment (Figure 6C,D).

### CEE Protected PC12 Cells Through the Regulation of JAK-STAT, HSP90 and DNA Damage/Telomere Stress Signaling Pathways

**FIGURE 7:**
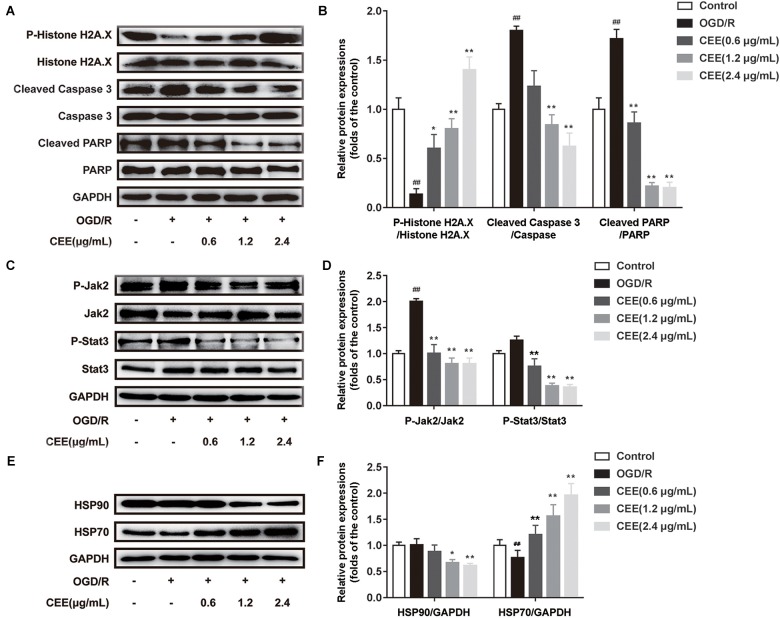
*Caesalpinia sappan* ethanolic extract protects PC12 cells through the regulation of JAK-STAT, HSP90 and DNA damage/telomere stress signaling pathways. **(A)** The expression of P-Histone H2A.X, Histone H2A.X, cleaved-PARP, PARP, cleaved-Caspase 3 and Caspase 3 as determined by western blotting. **(B)** Quantitation of the western blot analysis for P-Histone H2A.X compared with Histone H2A.X, cleaved-PARP compared with PARP, and cleaved-Caspase 3 compared with Caspase 3. **(C)** The expression of P-Jak2, Jak2, P-Stat3 and Stat3 as detected by western blotting. **(D)** Quantitation of the western blot analysis for P-Jak2 compared with Jak2, and P-Stat3 compared with Stat3. **(E)** The expression of HSP90 and HSP70 as determined by western blotting. **(F)** Quantitation of the western blot analysis for HSP90 compared with GAPDH, and HSP70 compared with GAPDH. Data are expressed as mean ±*SD*, *n* = 3. ^##^*P* < 0.01 vs. control group, ^∗∗^*P* < 0.01, ^∗^*P* < 0.05 vs. OGD/R group.

Based on previous bioinformatics analysis of the multiple targets of CEE, we further confirmed the effects of CEE on three major signaling pathways which were associated with JAK-STAT, HSP90, and DNA damage/telomere stress. Our results demonstrate that CEE could effectively inhibit the expression of cleaved-Caspase3 and cleaved-PARP, as well as enhancing the expression of P-Histone H2A.X (Figure 7A,B). These observations suggest that the caspase-dependent apoptotic pathway might be involved in the CEE-mediated neuroprotective effect. Additionally, CEE significantly decreased the ratio of P-Jak2/Jak2 and P-Stat3/Stat3 protein expression in the JAK-STAT signaling pathway (Figure 7C,D), which suggests that the inhibitory effect of CEE on neutrophil and microglial activation was exerted through the Jak2-Stat3 inflammation pathway. We also found that the expression of HSP90 was down-regulated and the expression of HSP70 was enhanced in PC12 cells stimulated by OGD/R following CEE treatment (Figure 7E,F), showing a regulatory effect on the molecular chaperones essential for helping proteins fold and limiting the dangerous aggregation of immature proteins.

## Discussion

In TCM, *C. sappan* L. exhibits great therapeutic potential, including the alleviation of dysmenorrhea, rheumatic pain and traumatic injuries and pain (Jia et al., 2014; Jung et al., 2015a). Previous pharmacological studies have demonstrated that *C. sappan* L. possesses immunomodulatory (Ye et al., 2006), anti-cancer (Raj et al., 2014), antioxidant (Saenjum et al., 2010), anti-inflammatory (Chu et al., 2013; Jung et al., 2015b; Tewtrakul et al., 2015) and sedative (Baek et al., 2000) activities. Notably, *C. sappan* L. has also been widely used for the treatment of ischemic cerebral stroke in the local populations of China (Xu and Lee, 2004; Wu et al., 2011; Islam et al., 2016; Jia et al., 2016). However, the detailed pharmacological mechanism of treatment still remains unknown.

The MCAO model has been used to demonstrate the protective effects of ursolic acid (Wang et al., 2018), l-homocarnosine (Huang et al., 2018) and Shengui Sansheng San (Liu et al., 2018). In the current study, our data demonstrates that CEE exerted neuroprotective effects both *in vitro* and *in vivo*. It is of note that the average mortality of MCAO operation is about 15%. CEE treatment caused less mortality in 30, 100, and 300 mg/kg treatment groups than the MCAO group; additionally, CEE treatment showed less mortality in 100 and 300 mg/kg groups than the GND group, indicating that CEE could exert a conspicuous neuroprotective effect against cerebral ischemia. Additionally, intraduodenal administration did not induce further death, suggesting that this process was not harmful to animals, at least in our study.

Neuronal apoptosis plays a major role in the pathological process of cerebral ischemic injury (Zille et al., 2012). Damaged mitochondria and DNA fragmentation are key markers for neuronal apoptosis (Cerqua et al., 2010). The TEM examinations show that CEE treatment obviously improved mitochondrial morphology and chromatin distribution. Thus, we speculate that CEE may suppress neuronal apoptosis by targeting mitochondria and DNA.

Ischemic stroke elicits a robust neuroinflammatory response that may contribute to neuronal cell death (Li et al., 2013). CD11b/c, a major integrin on neutrophil surface, is specifically expressed in the early stages of neutrophil migration into the inflammatory area. Activated neutrophils can release proinflammatory mediators which initiate a self-energizing cascade of proinflammation and destruction. Additionally, Iba-1 is a specific marker for microglia, which participate in the process of neuroinflammation (Morgan et al., 2008). Resveratrol attenuates the elevated expression of CD11b/c that is after cerebral ischemia (Girbovan and Plamondon, 2015). Tocovid is a new combination of tocotrienols and tocopherol (Jiao et al., 2018), and salvianolic acid B from the medicinal herb *Salvia miltiorrhiza* (Fan et al., 2018) downregulates the expression of the inflammatory markers ionized calcium binding adapter molecule-1 (Iba-1). Our data suggests that CEE treatment significantly decreases the expression of CD11b/c and Iba-1 in ischemic brains, finally resulting in an inhibitory effect on neuroinflammation likely through targeting the toll-like receptor or RHO GTPase signaling pathways.

In addition, inflammation is also the main reason for glial scar formation at the border between normal and ischemic tissue. The glial scar is one of the important factors accounting for the limited functional recovery from stroke (Hill et al., 2012). Astrocytes are the major glial cell type in the CNS (Choudhury and Ding, 2016), and the glial scar is mainly constituted by reactive astrocytes, along with associated extracellular matrix (ECM) proteins such as collagen. Our findings suggest that CEE may promote neuronal function recovery through the inhibition of astrocyte activation and collagen deposition in the MCAO model. Moreover, the glial scar is one of the main obstacles to axonal regeneration after cerebral injury. Microtubule associated protein 2 (MAP-2) is a marker of hypoxic-ischemic injury (Johnson and Jope, 1992; Crestini et al., 2001). Decreased expression of MAP-2 occurs in cerebral hypoxia (Oechmichen and Meissner, 2006).

Axonal regeneration is an important and complex process in the brain reorganization and repair that occurs after stroke (Burda and Sofroniew, 2014). In our study, we observed that the expression of MAP-2, an essential marker of axonal regeneration, was greatly up-regulated in CEE treated animals, suggesting that MAP-2 may play a pivotal role in the CEE-dependent protective effect on synapse morphology (Weng et al., 2011). Additionally, Nissl bodies are crucial for protein synthesis in the CNS (Jing et al., 2014). Our data demonstrates that the number of Nissl bodies in the neurons was significantly higher in the CEE treated animals than in the MCAO group, suggesting that it protected against MCAO-induced cerebral injury. Thus, CEE may potentially elicit neuroprotection by promoting axonal regeneration. There is increased survival of Nissl bodies after treatment with the neuroprotective agents borneol and ligustrazine and *Dioscorea zingiberensis* steroid saponins (Zhang et al., 2014; Yu et al., 2016). This finding seems to be closely related to our previous data, which indicated that protosappanin B (Zeng et al., 2015a), one of the major neuroprotective compounds in CEE, significantly increases the expression of the neurosynaptic markers GAP43 and MAP-2 in PC12 cells and primary neurons (Behravan et al., 2014).

In fact, the biological activities of the chemical constituents in herbs are decided by several factors. One is the relative net content of the constituent, and another is the pharmacological activity of the constituent itself. As for CEE, the chemical composition is highly complexed and associated with versatile biological functions. Some of the ingredients show obvious pharmacological effects on cell phenotype which can be detected easily; however, others may only help the drug transport or exert synergic action, which are difficult for activity valuation. Therefore, in this study, we are trying to find out the global target protein list which could show a general target landscape for the total constituents in CEE regardless of it exerting direct or indirect pharmacological effects.

Based on the use of small-molecule affinity chromatography technology, we successfully identified 150 potential target proteins of CEE. This technique permits the systematic study of the selectivity of pharmacological compounds and their expected possible adverse effects (Guiffant et al., 2007).

*C. sappan* ethanolic extract might directly interact with these target proteins to regulate neurobiological functions in the brain and further reverse the disease progression of cerebral ischemia. Moreover, it is worth mentioning that our observations revealed the direct bindings of CEE to different target proteins in neuronal cells. Among these proteins, some are kinases or protease, which are inhibited or activated by CEE; meanwhile, some CEE also appears to bind to structural proteins, which function only as scaffolding structures. Here, CEE binding does not lead to direct agonistic or antagonistic effects. Futhermore, we speculate that CEE might regulate the conformational changes of these proteins to further influence downstream protein functions as well as cell activity. Of course, these areas require in-depth investigation in the future.

The bioinformatics analysis, based on the identified targets, showed that CEE treatment influenced several biological signaling pathways, including JAK-STAT, HSP90, DNA damage/telomere stress, apoptotic execution and RHO GTPase. Our wet-lab experimental data demonstrated that CEE inhibited cell apoptosis by down-regulating the expression of cleaved caspase-3 and cleaved PARP, in addition to recovering DNA damage by inducing phosphorylation of H2A.X (Banerjee et al., 2017). Moreover, the aberrant activation of JAK-STAT signaling pathways may be responsible for the inflammatory response in ischemic stroke (De Zio et al., 2013). Our findings show that CEE inhibited JAK-STAT signaling pathway by down-regulating the phosphorylation of Jak2 and Stat3, leading to an inhibitory effect on microglial inactivation as well as neuroinflammation. Interestingly, we found that several compounds in CEE show effective anti-inflammatory actions, including protosappanin A, deoxysappanone B and sappanone A. 3-Deoxysappanchalcone (Kim et al., 2014), deoxysappanone B (Zeng et al., 2015b), sappanchalcone (Jung et al., 2015b), sappanone A (Jia et al., 2016), protosappanin A (Brouns and De Deyn, 2009), brazilin (Zeng et al., 2015a), episappanol, protosappanin C, brazilin, (iso-)protosappanin B, and sappanol (Mueller et al., 2016) are derived from *C. sappan* and all have anti-inflammatory activity. Particularly, protosappanin A could exert an anti-inflammatory effect through inhibition of the Jak2-Stat3 signaling pathway (Wang L.C. et al., 2017). Thus, we speculate that protosappanin A might be mainly involved in the CEE-mediated anti-inflammatory effects by targeting Jak2-Stat3.

Furthermore, the accumulation of misfolded proteins in the brain is one of the hallmarks of ischemic stroke. It has been found that molecular chaperones such as heat shock proteins (HSP70 and HSP90), which target misfolded proteins for ubiquitin-dependent degradation, are heavily involved in ischemic cerebral injury (Lackie et al., 2017). The titer of anti-Hsp antibodies increases in stroke (Banecka-Majkutewicz et al., 2014). Hsp 70 is downregulated in ischemic stroke (Demyanenko and Uzdensky, 2017). The neuroprotective effects of iodoacetate (Guo et al., 2001), geldanamycin (Kwon et al., 2008), and 17-allylamino-demethoxygeldanamycin (Li et al., 2015) are associated with an up-regulation of Hsp. Hsp70 downregulates dynamin which induces Fas–mediated apoptosis in the brain (Kim et al., 2016). Hsp70 has been proposed as a therapeutic target in stroke (Kim et al., 2018). We found that CEE treatment significantly increased the expression of HSP70, which is a key player in protein folding, cell repair and immune-regulation. Moreover, CEE decreased HSP90 expression, which might accelerate the degradation of misfolded proteins and inhibit collagen deposition after stroke. Because HSPs are widely activated in response to an accumulation of unfolded or misfolded proteins during stroke, we speculate that CEE treatment could show some regulatory effects specifically on stroke-related protein targets such as HSPs. However, these speculations require further investigation.

The herb *C. sappan* L. has been widely used in China for a long time with no reported cases of poisoning. Therefore, *C. sappan* L. has been recorded as non-toxic in the ancient Chinese medicinal books, such as the *Compendium of Materia Medica*. In our study, the potential toxicity of CEE was evaluated from four points, including mental state, food intake, fecal and urinary functions, and histopathology. We did not observe any obvious changes in the above indexes, suggesting that CEE is relatively safe, at least in the current dose range, for the neuronal, urinary and digestive systems.

Collectively, our study demonstrates that CEE can exert neuroprotective effects through the regulation of neuronal apoptosis, neuroinflammation and axonal generation. These biological processes may be controlled by the 150 identified potential target proteins that are associated with several signaling pathways including JAK-STAT, HSP90 and DNA damage/telomere stress. These findings show that CEE can exert neuroprotection through multi-targeted pharmacological mechanisms against ischemia/reperfusion-induced cerebral injury. *C. sappan* heartwood extract is devoid of both acute and subacute toxicity in male and female rats (Nirmal et al., 2015). Thus, CEE is a promising candidate for drug development.

## Author Contributions

K-WZ and P-FT designed the research. Y-JW conducted the majority of the experiments. LX and W-TS performed the rat MCAO model experiments. Y-QL and L-YL provided the CEE beads. M-BZ and YJ assisted in HPLC analysis. K-WZ, Y-JW, and L-CW wrote the manuscript.

## Conflict of Interest Statement

The authors declare that the research was conducted in the absence of any commercial or financial relationships that could be construed as a potential conflict of interest.

## Abbreviations

CCA: common carotid artery
CCD: charge-coupled device
CEE beads: CEE-conjugated solid beads
CEE: *C. sappan* L. ethanolic extract
CNS: central nervous system
ECA: external carotid artery
ECM: extracellular cell matrix
GFAP: glial fibrillary acidic protein
GND: Ginaton
GO BP: gene ontology biological process
Iba-1: ionized calcium binding adaptor molecule 1
ICA: internal carotid artery
IHC: immunohistochemistry
LSD: least significant difference
MAP-2: microtubule-associated protein-2
MCAO: middle cerebral artery occlusion
OGD/R: oxygen glucose deprivation followed by reperfusion
PBS: phosphate-buffered saline
PPA: pterygopalatine artery
PVDF: polyvinylidene fluoride
SD: Sprague-Dawley
SHR: HSP90 chaperone cycle for steroid hormone receptors
TCM: traditional Chinese medicine
TEM: transmission electron microscope
TTC: 2, 3, 5-triphenyltetrazolium chloride
TUNEL: TdT-mediated dUTP-biotin nick end labeling
UniProt: universal protein resource
