# Supplementary material for: The Ethanolic Extract of Caesalpinia sappan Heartwood Inhibits Cerebral Ischemia/Reperfusion Injury in a Rat Model Through a Multi-Targeted Pharmacological Mechanism
Source: Front Pharmacol. 2019 Feb 5;10:29. doi: 10.3389/fphar.2019.00029 (PMC6370896; doi:10.3389/fphar.2019.00029)
Supplement: Supplementary file 1 [file Data_Sheet_1.docx]

***Supplementary material***

**The ethanolic extract of *Caesalpinia sappan* heartwood inhibits cerebral ischemia/reperfusion injury in a rat model through a multi-targeted pharmacological mechanism**

Yan-Jun Wan^a^, Li Xu^b^, Wen-Ting Song^b^, Yu-Qi Liu^c^, Li-Chao Wang^a^, Ming-Bo Zhao^a^, Yong Jiang^a^, Lian-Ying Liu^c,^*, Ke-Wu Zeng^a,^*, Peng-Fei Tu^a,^*

^a^ State Key Laboratory of Natural and Biomimetic Drugs, School of Pharmaceutical Sciences, Peking University, Beijing 100191, China

^b^ Institute of Basic Medical Sciences, Xiyuan Hospital, China Academy of Chinese Medical Sciences, Beijing 100091, China

^c^ College of Materials Science and Engineering, Beijing University of Chemical Technology, Beijing 100029, China

Corresponding author at: State Key Laboratory of Natural and Biomimetic Drugs, School of Pharmaceutical Sciences, Peking University, Beijing 100191, China.

E-mail address: L.Y.L. (e-mail: lyliu@mail.buct.edu.cn), K.W.Z. (e-mail: ZKW@bjmu.edu.cn), P.F.T. (e-mail: pengfeitu@bjmu.edu.cn).

**Supporting Data**

**Supporting Table S1. 150 potential target binding proteins of CEE**

| Accession | Description | Gene Name |
| --- | --- | --- |
| P35579 | Myosin-9 | *MYH9* |
| P60709 | Actin, cytoplasmic 1 | *ACTB* |
| P68133 | Actin, alpha skeletal muscle | *ACTA1* |
| Q86YZ3 | Hornerin | *HRNR* |
| P68032 | Actin, alpha cardiac muscle 1 | *ACTC1* |
| P22626 | Heterogeneous nuclear ribonucleoproteins A2/B1 | *HNRNPA2B1* |
| Q8NC51 | Plasminogen activator inhibitor 1 RNA-binding protein | *SERBP1* |
| P61978-3 | Isoform 3 of Heterogeneous nuclear ribonucleoprotein K | *HNRNPK* |
| P60660-2 | Isoform Smooth muscle of Myosin light polypeptide 6 | *MYL6* |
| Q562R1 | Beta-actin-like protein 2 | *ACTBL2* |
| P11021 | 78 kDa glucose-regulated protein | *HSPA5* |
| P51991 | Heterogeneous nuclear ribonucleoprotein A3 | *HNRNPA3* |
| P62269 | 40S ribosomal protein S18 | *RPS18* |
| Q02413 | Desmoglein-1 | *DSG1* |
| P15924 | Desmoplakin | *DSP* |
| P62917 | 60S ribosomal protein L8 | *RPL8* |
| J3QR09 | Ribosomal protein L19 | *RPL19* |
| P04908 | Histone H2A type 1-B/E | *HIST1H2AB* |
| P20671 | Histone H2A type 1-D | *HIST1H2AD* |
| P26373 | 60S ribosomal protein L13 | *RPL13* |
| P23527 | Histone H2B type 1-O | *HIST1H2BO* |
| P58876 | Histone H2B type 1-D | *HIST1H2BD* |
| P09429 | High mobility group protein B1 | *HMGB1* |
| Q13442 | 28 kDa heat- and acid-stable phosphoprotein | *PDAP1* |
| P10412 | Histone H1.4 | *HIST1H1E* |
| Q92841 | Probable ATP-dependent RNA helicase DDX17 | *DDX17* |
| P68104 | Elongation factor 1-alpha 1 | *EEF1A1* |
| P35749-4 | Isoform 4 of Myosin-11 | *MYH11* |
| P13646 | Keratin, type I cytoskeletal 13 | *KRT13* |
| P14923 | Junction plakoglobin | *JUP* |
| P07437 | Tubulin beta chain | *TUBB* |
| P11142 | Heat shock cognate 71 kDa protein | *HSPA8* |
| P62701 | 40S ribosomal protein S4, X isoform | *RPS4X* |
| B4DLW8 | Probable ATP-dependent RNA helicase DDX5 | *DDX5* |
| P18124 | 60S ribosomal protein L7 | *RPL7* |
| F8W6I7 | Heterogeneous nuclear ribonucleoprotein A1 | *HNRNPA1* |
| P68371 | Tubulin beta-4B chain | *TUBB4B* |
| P61247 | 40S ribosomal protein S3a | *RPS3A* |
| P23246 | Splicing factor, proline- and glutamine-rich | *SFPQ* |
| P02545 | Prelamin-A/C | *LMNA* |
| P06753-2 | Isoform 2 of Tropomyosin alpha-3 chain | *TPM3* |
| P35580 | Myosin-10 | *MYH10* |
| P06753-5 | Isoform 5 of Tropomyosin alpha-3 chain | *TPM3* |
| Q13885 | Tubulin beta-2A chain | *TUBB2A* |
| P11940 | Polyadenylate-binding protein 1 | *PABPC1* |
| Q86V81 | THO complex subunit 4 | *ALYREF* |
| Q15056-2 | Isoform Short of Eukaryotic translation initiation factor 4H | *EIF4H* |
| E9PCY7 | Heterogeneous nuclear ribonucleoprotein H | *HNRNPH1* |
| P62081 | 40S ribosomal protein S7 | *RPS7* |
| Q5D862 | Filaggrin-2 | *FLG2* |
| P39019 | 40S ribosomal protein S19 | *RPS19* |
| P42766 | 60S ribosomal protein L35 | *RPL35* |
| P08238 | Heat shock protein HSP 90-beta | *HSP90AB1* |
| P62979 | Ubiquitin-40S ribosomal protein S27a | *RPS27A* |
| P06753-6 | Isoform 6 of Tropomyosin alpha-3 chain | *TPM3* |
| Q9Y3U8 | 60S ribosomal protein L36 | *RPL36* |
| Q13283 | Ras GTPase-activating protein-binding protein 1 | *G3BP1* |
| P62987 | Ubiquitin-60S ribosomal protein L40 | *UBA52* |
| P07355 | Annexin A2 | *ANXA2* |
| Q9P258 | Protein RCC2 | *RCC2* |
| P01857 | Ig gamma-1 chain C region | *IGHG1* |
| Q71U36-2 | Isoform 2 of Tubulin alpha-1A chain | *TUBA1A* |
| P62424 | 60S ribosomal protein L7a | *RPL7A* |
| J3KN67 | Tropomyosin alpha-3 chain | *TPM3* |
| Q15233 | Non-POU domain-containing octamer-binding protein | *NONO* |
| M0R0F0 | 40S ribosomal protein S5 (Fragment) | *RPS5* |
| H0YK48 | Tropomyosin alpha-1 chain | *TPM1* |
| K7EM56 | 40S ribosomal protein S15 | *RPS15* |
| P62263 | 40S ribosomal protein S14 | *RPS14* |
| Q9Y3Y2-4 | Isoform 3 of Chromatin target of PRMT1 protein | *CHTOP* |
| P08708 | 40S ribosomal protein S17 | *RPS17* |
| Q14157 | Ubiquitin-associated protein 2-like | *UBAP2L* |
| H7BYY1 | Tropomyosin 1 (Alpha), isoform CRA_m | *TPM1* |
| P62851 | 40S ribosomal protein S25 | *RPS25* |
| P08670 | Vimentin | *VIM* |
| P35637-2 | Isoform Short of RNA-binding protein FUS | *FUS* |
| P06576 | ATP synthase subunit beta, mitochondrial | *ATP5B* |
| P25705 | ATP synthase subunit alpha, mitochondrial | *ATP5A1* |
| P23396 | 40S ribosomal protein S3 | *RPS3* |
| P46781 | 40S ribosomal protein S9 | *RPS9* |
| P02788-2 | Isoform DeltaLf of Lactotransferrin | *LTF* |
| P63000 | Ras-related C3 botulinum toxin substrate 1 | *RAC1* |
| P07951-2 | Isoform 2 of Tropomyosin beta chain | *TPM2* |
| P0C0S5 | Histone H2A.Z | *H2AFZ* |
| P10809 | 60 kDa heat shock protein, mitochondrial | *HSPD1* |
| P07951 | Tropomyosin beta chain | *TPM2* |
| P46777 | 60S ribosomal protein L5 | *RPL5* |
| P38159 | RNA-binding motif protein, X chromosome | *RBMX* |
| P68366-2 | Isoform 2 of Tubulin alpha-4A chain | *TUBA4A* |
| Q8IUE6 | Histone H2A type 2-B | *HIST2H2AB* |
| Q14444-2 | Isoform 2 of Caprin-1 | *CAPRIN1* |
| P30050 | 60S ribosomal protein L12 | *RPL12* |
| P62805 | Histone H4 | *HIST1H4A* |
| P62750 | 60S ribosomal protein L23a | *RPL23A* |
| P46779 | 60S ribosomal protein L28 | *RPL28* |
| P81605 | Dermcidin | *DCD* |
| P62277 | 40S ribosomal protein S13 | *RPS13* |
| O00571-2 | Isoform 2 of ATP-dependent RNA helicase DDX3X | *DDX3X* |
| P16401 | Histone H1.5 | *HIST1H1B* |
| P67809 | Nuclease-sensitive element-binding protein 1 | *YBX1* |
| Q00839-2 | Isoform Short of Heterogeneous nuclear ribonucleoprotein U | *HNRNPU* |
| Q9Y295 | Developmentally-regulated GTP-binding protein 1 | *DRG1* |
| P61626 | Lysozyme C | *LYZ* |
| Q07020 | 60S ribosomal protein L18 | *RPL18* |
| Q6UWP8 | Suprabasin | *SBSN* |
| Q8WXF1 | Paraspeckle component 1 | *PSPC1* |
| P62857 | 40S ribosomal protein S28 | *RPS28* |
| P46783 | 40S ribosomal protein S10 | *RPS10* |
| E5RI99 | 60S ribosomal protein L30 (Fragment) | *RPL30* |
| P29508 | Serpin B3 | *SERPINB3* |
| O43684-2 | Isoform 2 of Mitotic checkpoint protein BUB3 | *BUB3* |
| P82979 | SAP domain-containing ribonucleoprotein | *SARNP* |
| Q9Y3I0 | tRNA-splicing ligase RtcB homolog | *RTCB* |
| P61353 | 60S ribosomal protein L27 | *RPL27* |
| P01876 | Ig alpha-1 chain C region | *IGHA1* |
| Q5T749 | Keratinocyte proline-rich protein | *KPRP* |
| P14625 | Endoplasmin | *HSP90B1* |
| P01834 | Ig kappa chain C region | *IGKC* |
| D3YTB1 | 60S ribosomal protein L32 (Fragment) | *RPL32* |
| P06702 | Protein S100-A9 | *S100A9* |
| P04406 | Glyceraldehyde-3-phosphate dehydrogenase | *GAPDH* |
| P36578 | 60S ribosomal protein L4 | *RPL4* |
| Q14847 | LIM and SH3 domain protein 1 | *LASP1* |
| Q9BUF5 | Tubulin beta-6 chain | *TUBB6* |
| M0R2L9 | 40S ribosomal protein S19 (Fragment) | *RPS19* |
| P23588 | Eukaryotic translation initiation factor 4B | *EIF4B* |
| P20700 | Lamin-B1 | *LMNB1* |
| P07900 | Heat shock protein HSP 90-alpha | *HSP90AA1* |
| Q96AG4 | Leucine-rich repeat-containing protein 59 | *LRRC59* |
| P54652 | Heat shock-related 70 kDa protein 2 | *HSPA2* |
| P17096 | High mobility group protein HMG-I/HMG-Y | *HMGA1* |
| P67936-2 | Isoform 2 of Tropomyosin alpha-4 chain | *TPM4* |
| B4DVY1 | Eukaryotic translation initiation factor 3 subunit D | *EIF3D* |
| P60866 | 40S ribosomal protein S20 | *RPS20* |
| P60842 | Eukaryotic initiation factor 4A-I | *EIF4A1* |
| Q08554-2 | Isoform 1B of Desmocollin-1 | *DSC1* |
| P62249 | 40S ribosomal protein S16 | *RPS16* |
| P25311 | Zinc-alpha-2-glycoprotein | *AZGP1* |
| P06733 | Alpha-enolase | *ENO1* |
| Q5VY30 | Retinol binding protein 4, plasma, isoform CRA_b | *RBP4* |
| Q92499 | ATP-dependent RNA helicase DDX1 | *DDX1* |
| P50454 | Serpin H1 | *SERPINH1* |
| P61313 | 60S ribosomal protein L15 | *RPL15* |
| Q01844-6 | Isoform 6 of RNA-binding protein EWS | *EWSR1* |
| P31944 | Caspase-14 | *CASP14* |
| P31025 | Lipocalin-1 | *LCN1* |
| Q96PK6 | RNA-binding protein 14 | *RBM14* |
| P05109 | Protein S100-A8 | *S100A8* |
| P38646 | Stress-70 protein, mitochondrial | *HSPA9* |


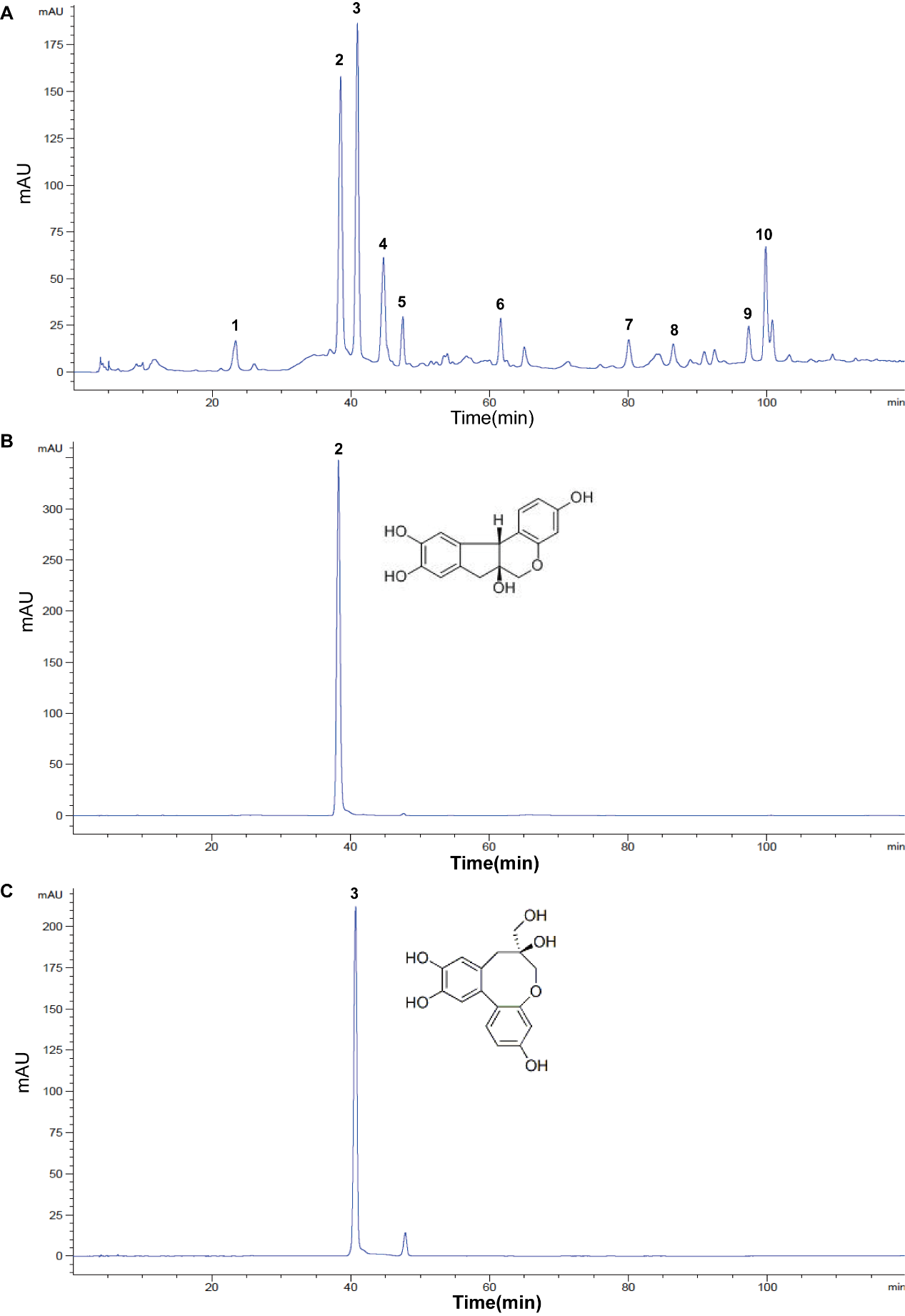


**Supporting Figure S1. HPLC profile of *Caesalpinia sappan* L. heartwood ethanol extract (CEE) and standards.**

(A) Chromatogram of *Caesalpinia sappan* L. heartwood ethanol extract (CEE). (B) Chromatogram of brazilian. (C) Chromatogram of protosappanin B.

Note: The letters 2 and 3 indicate the peaks corresponding to brazilian and protosappanin B, respectively.

**Extended Experimental Procedure**

- 1. **Preparation of the beads-coupled to CEE**

Firstly, thiol-functionalized Fe_3_O_4_ nanoparticles were synthesized using a modified (Fe^3+^/Fe^2+^) co-precipitation method. Briefly, FeSO_4_·7H_2_O (0.35 g), FeCl_3_·6H_2_O (0.32 g), HS-PEG1k-SH (0.1 g, synthesized following the procedure outlined by Lundberg P., *et al.* (Lundberg et al., 2011)) and H_2_O (30 mL) were added into a 100 mL three-neck flask and mechanically stirred under an atmosphere of N_2_. The solution was heated to 70°C and stabilized for 30 min. Then, NH_3_·H_2_O (4 mL, 25% wt) was quickly added, and the reaction was carried out for 2 h. The as-synthesized Fe_3_O_4_ NPs (Fe_3_O_4_-PEG-SH NPs) were magnetically separated, washed with deionized water and methanol (MeOH) at least 3 times, and then dispersed in MeOH for further use.

Secondly, Fe_3_O_4_-PEG-SH NPs (0.05 g), 4-acryloyloxybenzophenone (ABP, 0.01 g, synthesized according to a previously described procedure (Wang et al., 2013)), triethylamine (TEA, 30 μL) and acetone (20 mL) were added into a 100 mL three-neck round-bottom flask equipped with a mechanical stirrer and a condenser, and mechanically stirred for 3 h under an atmosphere of N_2_ at room temperature. The resultant Fe_3_O_4_ NPs were detected with SEM (Fig.S2) and FT-IR (Fig.S3). FT-IR analyses indicated that the characteristic absorptions of ABP at 1734 cm^-1^ and 1300-1500 cm^-1^. The Fe_3_O_4_ NPs were centrifuged, washed with acetone and MeOH at least 3 times, and then dispersed in MeOH for further use.





**Fig S2.** SEM image of Fe_3_O_4_-PEG-ABP NPs





**Fig S3.** FT-IR spectra of Fe_3_O_4_-PEG-ABP NPs, Fe_3_O_4_-PEG-SH NPs and monomer ABP

Lastly, Fe_3_O_4_-PEG-ABP NPs (0.01 g), CEE (0.01 g) and H_2_O (20 mL) were placed in a special cylindrical quartz reactor (Φ 5 cm, H 9 cm, with a quartz cap, N_2_ inlet and outlet), bubbled with N_2_ for 30 min_,_ and then exposed to UV light (375 W high-pressure mercury lamp, incident light intensity 12.5 W/m^2^, λ = 254 nm) for 1 h. The obtained Fe_3_O_4_ NPs (Fe_3_O_4_-PEG-ABP-CEE NPs) were magnetically separated and washed with deionized water at least 3 times. The characteristic absorption of CEE at around 284 nm appeared in UV-vis spectrum of Fe_3_O_4_-PEG-ABP-CEE NPs (Fig. S4), indicating the coupling of CEE on Fe_3_O_4_ NPs.





**Fig S4.** UV-vis spectra of Fe_3_O_4_-PEG-ABP NPs, Fe_3_O_4_-PEG-ABP-CEE NPs, ABP and CEE.

**Supporting References**

Lundberg, P., Walter, M. V., Montanez, M. I., Hult, D., Hult, A., Nystrom, A., et al. (2011). Linear dendritic polymeric amphiphiles with intrinsic biocompatibility: synthesis and characterization to fabrication of micelles and honeycomb membranes. *Polym. Chem-Uk*. 2, 394-402. doi:10.1039/c0py00258e.

Wang, S. L., Yue, K., Liu, L. Y., Yang, W. T. (2013). Photoreactive, core-shell cross-linked/hollow microspheres prepared by delayed addition of cross-linker in dispersion polymerization for antifouling and immobilization of protein. *J. Colloid. Interf. Sci.* 389, 126-133. doi:10.1016/j.jcis.2012.08.047.
